# Echocardiography and laboratory outcomes of COVID-19 in children with a history of Kawasaki disease: a preliminary observation

**DOI:** 10.3389/fcvm.2023.1127892

**Published:** 2023-10-04

**Authors:** Mindy Ming-Huey Guo, Ling-Sai Chang, Yu-Jhen Chen, Ho-Chang Kuo

**Affiliations:** ^1^ Kawasaki Disease Center, Kaohsiung Chang Gung Memorial Hospital, Kaohsiung, Taiwan; ^2^ Department of Pediatrics, Kaohsiung Chang Gung Memorial Hospital, Kaohsiung, Taiwan; ^3^ College of Medicine, Chang Gung University, Taoyuan, Taiwan; ^4^ School of Medicine, Chung Shan Medical University, Taichung, Taiwan; ^5^ Department of Respiratory Therapy, Kaohsiung Chang Gung Memorial Hospital, Kaohsiung, Taiwan

**Keywords:** Kawasaki disease, COVID-19, echocardiography, vaccine, coronary artery lesions

## Abstract

**Background:**

Infection with SARS-CoV-2 virus has been associated with cardiovascular sequelae including multisystem inflammatory syndrome (MIS-C) in children. Patients with a prior history of Kawasaki disease, may be more susceptible to changes in echocardiographic or laboratory findings after COVID-19. The objective of this study was to investigate the echocardiographic and laboratory findings in children with a prior history of Kawasaki disease after SARS-CoV-2 infection.

**Materials and methods:**

In this study, we performed a retrospective chart review of 41 children younger than 18 years old who were diagnosed with COVID-19 from April to August of 2022 and had a prior history KD. We included echocardiography and blood draw data obtained at the last outpatient follow-up at our hospital for KD, and within 4 months of SARS-CoV-2 infection. Echocardiographic data obtained from 82 age-matched and gender matched controls were also included for comparison.

**Results:**

We found that COVID-19 resulted in slightly higher RCA *Z*-scores within the first month after infection (mean ± SE, 1.20 ± 0.18 vs. 0.83 ± 0.18, *p* = 0.030), although this increase did not result in coronary artery dilatation, defined as a *Z*-score of at least 2.5. In addition, we found that degree of RCA dilatation after COVID-19 infection was negatively correlated with the change in monocyte percentage (Pearson's correlation coefficient—0.363, *p* = 0.020). Moreover, RCA *Z*-score changes were lower in patients who received at least one dose of mRNA COVID-19 vaccine when compared those who did not receive any (mean ± SE, −0.23 ± 0.16 vs. 0.39 ± 0.17, *p* = 0.031).

**Conclusion:**

In this pilot study we found that COVID-19 infection resulted in slightly higher RCA *Z*-scores in children with a prior history of KD, although not large enough to be classified as coronary aneurysms. While these changes could be the result of measurement imprecision or interobserver variation, further study of the cardiac outcomes of COVID-19 infection in children with a prior history of KD are needed in the future.

## 1. Introduction

Kawasaki disease (KD) is a childhood systemic vasculitis that effects the small to medium size vessels. Common complications are mostly cardiovascular, including coronary artery dilatations and aneurysms (1). Coronavirus disease-2019 (COVID-19), which is caused by the coronary virus SARS-CoV-2, and is responsible for a global pandemic, has mostly been associated with respiratory outcomes. However, there have been reports that COVID-19 may also cause myocarditis, myocardial infarction, arrhythmia and other cardiovascular sequalae (2) in up to 2%–4% of COVID patients (3). Mechanisms of injury may be caused by direct damage of SARS-CoV-2 viral invasion of cardiomyocytes by binding to angiotensin-converting enzyme 2 (ACE2), resulting in the downregulation of ACE2, and overactivity of the renin angiotensin pathway. Cardiovascular injury may also be the result of indirect mechanisms including immunological injury from an inflammatory cytokine storm or activation of cellular immunity (2). In adult patients, increased mortality due to myocarditis after SARS-COV-2 infection was higher in patient with underlying cardiovascular disease (4). In children, Multisystem inflammatory syndrome in children (MIS-C) is a recently recognized disease entity that occurs 2–6 weeks after COVID-19 infection. There is considerable overlap of symptoms of MIS-C and KD, as both conditions may cause fever, mucocutaneous inflammatory such as oral erythema and strawberry tongue, and myocardial dysfunction, in fact around 40% of children with MIS-C also have Kawasaki disease-like clinical features (5).

There is growing interest regarding clinical outcomes of COVID-19 in patients with a history of autoimmune diseases such as rheumatic arthritis or systemic lupus erythematosus. Data extracted from the COVID-19 Global Rheumatology Alliance physician-reported registry found that patients with rheumatic arthritis were likely to have severe COVID requiring hospitalization (6). Less is known about outcomes of COVID-19 in children with a history of Kawasaki disease. In a study which used electronic medical records obtained from Washington State, in the United States, COVID-19 did not result in higher rates of hospitalization or severe disease in 37 patients with a history of Kawasaki disease (7). However, this study did not include echocardiographic or laboratory data. Given that COVID-19 has been associated with cardiovascular complications and MIS-C, we performed a retrospective review of echocardiography and laboratory data after COVID-19 in children with a prior history of KD.

## 2. Materials and methods

Taiwan witnessed a minimal number of COVID-19 cases throughout 2020 and 2021, with only small pockets of outbreak sporadically. The Omicron wave, which emerged in Taiwan in March in 2022 (8) was the first large scale outbreak in Taiwan, with over 50,000 the number of confirmed cases daily according to Taiwan's Centers for Disease Control (9). In this study, we performed a retrospective chart review of 41 children younger than 18 years old who were diagnosed with COVID-19 from April to August of 2022 and had a prior history KD. In addition, the echocardiographic data of 82 age-matched, gender-matched controls who were enrolled as healthy controls in our Kawasaki disease research cohort spanning from September 2021 to September of 2022. Echocardiography from healthy controls were specifically obtained for the purpose of comparison with patients with Kawasaki disease, and *Z*-scores of the coronary arteries were collected at the time of imaging. All patients included in this study received regular out-patient follow-up for KD at our hospital, a tertiary care center in southern Taiwan. COVID-19 was confirmed if the patient presented with fever, upper respiratory or abdominal symptoms along with either a positive COVID-19 rapid antigen test or if SARS-CoV-2 was detected via polymerase chain reaction. Patients were diagnosed with KD if they fulfilled the criteria set forth by the American Heart Association. To reiterate, the criteria mentioned includes fever for 5 days or more, and at least 4 out of the 5 following symptoms: rash, swelling of the palms or soles, lymph node swelling, conjunctivitis or redness, or peeling of the oral mucosa. Patients were also diagnosed with KD if they presented with at least two or three of the classical symptoms of KD, and fulfilled additional laboratory data or echocardiography findings according to criteria outlined by the American Heart Association (1).

Data was obtained by retrospective chart review and include echocardiography and blood draw data obtained at the last outpatient follow-up at our hospital for KD, and within 4 months of COVID-19 infection. Echocardiographic data included the diameter of the left coronary artery (LCA), left anterior descending artery (LAD), right coronary artery (RCA) and left ventricular ejection fraction (LVEF). The diameter of the coronary arteries was then converted into *Z*-scores according to height and weight using data provided by Taiwan Society of Pediatric Cardiology (10). Patients were determined as having coronary artery lesions if the inner diameter of the coronary arteries had a calculated *Z*-score of >2.5, in accordance with recommendations set forth by in the American Heart Association (1). Laboratory data collected included white blood cells counts, differential count, platelet count, hemoglobulin, aspartate aminotransferase (AST), alanine aminotransferase (ALT) and C-reactive protein levels (CRP). Demographic data collected included sex, age at KD diagnosis, age at COVID-19 diagnosis, and number of COVID-19 vaccines received.

Statistical analysis was performed using SPSS Version 20 (Chicago, USA). Paired *t*-test was used to compare coronary artery *Z*-scores and laboratory data before and after COVID-19 infection. Student's *t*-test was used to compare changes in coronary artery *Z*-scores and laboratory data after COVID-19 infection in children who did or did not receive COVID-19 mRNA vaccination. Pearson's correlation was used to determine whether there was a correlation between coronary artery values and other echocardiographic, laboratory or demographic data. A *p*-value of less than 0.05 was considered significant.

## 3. Results

In total 41 patients were included in this study, of which 27 patients were male, and 13 received at least one dose of a mRNA COVID-19 vaccine. Average age at the time of KD diagnosis was 2.03 ± 0.26 years (mean ± SE). Average age at the time of COVID-19 diagnosis was 7.89 ± 0.68 years (mean ± SE). COVID-19 was diagnosed 5.86 ± 0.65 years (mean ± SE) on average, after KD diagnosis. All 41 children with prior KD in our study presented with mild upper respiratory symptoms of COVID-19. There were no instances of lower respiratory tract infections, or cardiac complication such as myocarditis or MIS-C. Of the 41 patients enrolled in our study, 5 patients had coronary artery aneurysms in the subacute phase of KD, and only 2 patients had persistent coronary artery aneurysms prior to COVID-19 infection. There was no significant difference in laboratory data including white blood cell count, differential, platelet count, hemoglobulin, AST, ALT or CRP levels before or after COVID-19 infection (Table 1). However, patients had slightly higher RCA *Z*-scores after COVID-19 infection (mean ± SE, 1.20 ± 0.18 vs. 0.83 ± 0.18, *p* = 0.030), although this increase did not result in coronary artery dilatation (defined as a *Z*-score of at least 2.5). There was no significant difference in LCA, LAD *Z*-scores or LVEF percentage before or after COVID-19 infection. In addition, we found that the duration between KD diagnosis and COVID-19 infection were not associated with changes echocardiography or laboratory data with the exception of ALT (Pearson's correlation 0.895, *p* = 0.003). However, because only 8 out of the 41 patients included in our review had adequate ALT data for comparison, it is difficult to determine the significance of this correlation. Of note, the average ALT levels before and after COVID infection were both within normal range (Table 1).

**Table 1:** Comparison of coronary artery *Z*-scores and laboratory data before and after COVID-19 infection.

	Before COVID-19 (mean ± SE)	After COVID-19 (mean ± SE)	*p*-value
White blood cell count (10^9^/L)	6.99 ± 0.36 (*n* = 41)	7.03 ± 0.37 (*n* = 41)	0.930
Hemoglobulin (g/dl)	12.60 ± 0.15 (*n* = 41)	12.70 ± 0.15 (*n* = 41)	0.406
Platelet count (10^9^/L)	315.71 ± 12.02 (*n* = 41)	318.78 ± 12.39 (*n* = 41)	0.751
Segment (%)	41.50 ± 1.76 (*n* = 41)	44.71 ± 1.90 (*n* = 41)	0.059
Lymphocyte (%)	47.86 ± 1.67 (*n* = 41)	45.13 ± 1.80 (*n* = 41)	0.102
Monocyte (%)	5.69 ± 0.33 (*n* = 41)	5.87 ± 0.27 (*n* = 41)	0.684
Eosinophil (%)	4.04 ± 0.45 (*n* = 41)	3.57 ± 0.35 (*n* = 41)	0.159
Basophil (%)	0.65 ± 0.08 (*n* = 41)	0.63 ± 0.07 (*n* = 41)	0.824
Aspartate transaminase (U/L)	32.62 ± 1.73 (*n* = 8)	32.75 ± 2.12 (*n* = 8)	0.945
Alanine transaminase (U/L)	18.89 ± 1.32 (*n* = 9)	19.00 ± 2.63 (*n* = 9)	0.961
C-reactive protein (mg/L)	9.97 ± 9.70 (*n* = 9)	0.20 ± 0.00 (*n* = 9)	0.344
Left coronary artery (*Z*-score)	0.78 ± 0.20 (*n* = 41)	0.90 ± 0.21 (*n* = 41)	0.405
Left anterior descending artery (*Z*-score)	0.75 ± 0.09 (*n* = 40)	0.91 ± 0.06 (*n* = 40)	0.062
Right coronary artery (*Z*-score)	0.83 ± 0.18 (*n* = 41)	1.20 ± 0.18 (*n* = 41)	0.030*
Left ventricular ejection fraction (%)	65.69 ± 0.97 (*n* = 35)	62.89 ± 0.92 (*n* = 35)	0.053

* Denotes a *p*-value of less than 0.05.

**Figure 1:**
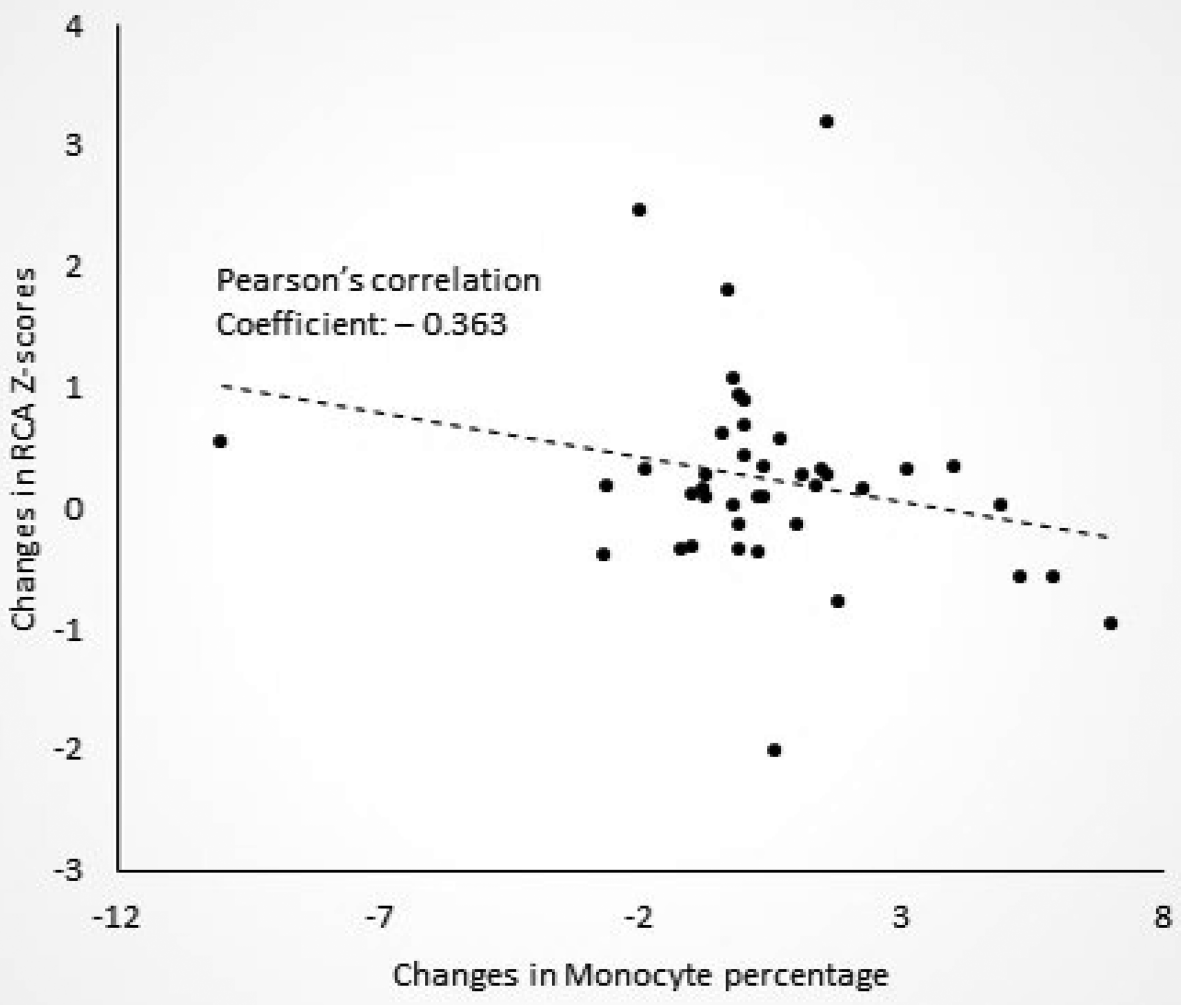
Correlation between changes in right coronary artery (RCA) *Z*-scores and changes in monocyte percentages after COVID-19 in patients with a history of Kawasaki disease.

We then tried to determine whether the extent of RCA dilatation was associated with changes in laboratory data or LCA or LAD *Z*-scores. We found that degree of RCA dilatation after COVID-19 infection was negatively correlated with the change in monocyte percentage (Pearson's correlation coefficient—0.363, *p* = 0.020, Figure 1). In other words, the patients with a larger RCA *Z*-scores after COVID-19 infection, were more likely to have decreased monocyte percentages on blood draw when compared to before COVID-19 infection. There was no correlation between the change in RCA *Z*-scores with age of KD or COVID-19 infection, or changes in laboratory data or LCA or LAD *Z*-scores before and after COVID-19 infection.

We also compared the changes in coronary artery diameter and laboratory data before and after COVID-19 infection in patients who did or did not receive mRNA COVID-19 vaccination. Changes in laboratory data and echocardiography finding were calculated as follows: value after COVID-19 infection minus value before COVID-19 infection (Table 2), which we felt were a better representation of changes in coronary artery diameter over time, and would account for patients who had larger coronary artery diameters at baseline. We found that changes in RCA *Z*-score after COVID-19 infection was slightly lower in patients who received at least one dose of mRNA COVID-19 vaccine when compared those who did not receive any doses (mean ± SE, −0.23 ± 0.16 vs. 0.39 ± 0.17, *p* = 0.031) (Table 2). Of note, there were two patients with coronary artery aneurysms at the time of COVID infection were in the vaccinated group, one of which had a RCA *Z*-score of 5.88 which increased to 6.07 after COVID infection, the other which had a RCA *Z*-score of 4.09 which decreased to 3.76 after COVID infection. There is a possibility that these changes may be the result of interobserver variation or measurement imprecision. There was no significant difference in the other echocardiography or laboratory data between the two groups.

**Table 2:** Changes in coronary artery *Z*-scores and laboratory values after COVID-19 infection in patients with and without COVID-19 vaccination.

	No vaccine (mean ± SE)	Vaccine (mean ± SE)	*p*-value
White blood cell count (10^9^/L)	0.54 ± 0.55 (*n* = 28)	0.11 ± 0.54 (*n* = 13)	0.632
Hemoglobulin (g/dl)	0.54 ± 0.05 (*n* = 28)	0.16 ± 0.21 (*n* = 13)	0.626
Platelet count (10^9^/L)	10 ± 18.91 (*n* = 28)	7.23 ± 7.75 (*n* = 13)	0.923
Segment (%)	6.76 ± 2.78 (*n* = 28)	1.72 ± 2.43 (*n* = 13)	0.261
Lymphocyte (%)	−2.87 ± 2.18 (*n* = 28)	−2.44 ± 2.23 (*n* = 13)	0.904
Monocyte (%)	0.46 ± 0.59 (*n* = 28)	0.50 ± 0.51 (*n* = 13)	0.970
Eosinophil (%)	−0.67 ± 0.46 (*n* = 28)	0.52 ± 0.48 (*n* = 13)	0.121
Basophil (%)	0.07 ± 0.14 (*n* = 28)	−0.15 ± 0.11 (*n* = 13)	0.330
Aspartate transaminase (U/L)	−1.17 ± 1.66 (*n* = 6)	4.00 ± 5.00 (*n* = 2)	0.227
Alanine transaminase (U/L)	−1.33 ± 1.43 (*n* = 6)	8.00 ± 7.00 (*n* = 2)	0.403
C-reactive protein (mg/L)	−0.08 ± 0.08 (*n* = 6)	0.00 ± 0.00 (*n* = 2)	0.603
Left coronary artery (*Z*-score)	0.21 ± 0.20 (*n* = 28)	0.31 ± 0.24 (*n* = 13)	0.784
Left anterior descending artery (*Z*-score)	0.22 ± 0.09 (*n* = 28)	0.23 ± 0.19 (*n* = 12)	0.934
Right coronary artery (*Z*-score)	0.39 ± 0.17 (*n* = 28)	−0.23 ± 0.16 (*n* = 13)	0.031*
Left ventricular ejection fraction (%)	−2.44 ± 1.59 (*n* = 23)	−3.58 ± 2.78 (*n* = 12)	0.704

* Denotes a *p*-value of less than 0.05.

We also collected a separate cohort of 81 age-matched and gender matched healthy controls, 41 of which were collected from September 2021 to February 2022 (age mean ± SE, 8.62 ± 0.27 years, 27 male), and 41 of where were collected from March 2022 to September 2022 (age mean ± SE, 8.11 ± 0.39 years, 27 male). We found that there were no significant differences in echocardiographic measurements of the LCA, LAD or RCA *Z*-scores in the period before (September 2021 to February 2022) and after (March 2022 to September 2022) the emergence of the Omicron variant in Taiwan (Table 3). We also compared healthy controls prior to the Omicron wave (September 2021 to February 2022) with KD patients prior to COVID infection, and found that patients with KD had statistically higher LCA, LAD and RCA *Z*-scores when compared to healthy controls within the same time period (Table 3). Likewise, we compared healthy controls after the Omicron wave (March 2022 to September 2022) with KD patients after COVID infection and found that, again, KD patients had higher LCA, LAD and RCA *Z*-scores when compared to healthy controls within the same time period (Table 3).

**Table 3:** Comparison of echocardiographic *Z*-scores in healthy children before and after March 2022.

	Healthy controls before March 2022^a^ (mean ± SE)	Healthy controls after March 2022^b^ (mean ± SE)	*p*-value
Left coronary artery (*Z*-score)	0.019 ± 0.137 (*n* = 41)	−0.118 ± 0.086 (*n* = 41)	0.398
Left anterior descending artery (*Z*-score)	0.205 ± 0.091 (*n* = 41)	0.379 ± 0.086 (*n* = 41)	0.170
Right coronary artery (*Z*-score)	0.246 ± 0.181 (*n* = 41)	0.344 ± 0.076 (*n* = 41)	0.619
	Healthy controls before March 2022^a^ (mean ± SE)	Kawasaki disease patients before COVID (mean ± SE)	*p*-value
Left coronary artery (*Z*-score)	0.019 ± 0.137 (*n* = 41)	0.78 ± 0.20 (*n* = 41)	0.005
Left anterior descending artery (*Z*-score)	0.205 ± 0.091 (*n* = 41)	0.75 ± 0.09 (*n* = 40)	0.001
Right coronary artery (*Z*-score)	0.246 ± 0.181 (*n* = 41)	0.83 ± 0.18 (*n* = 41)	0.019
	Healthy controls after March 2022^b^ (mean ± SE)	Kawasaki disease patients after COVID (mean ± SE)	*p*-value
Left coronary artery (*Z*-score)	−0.118 ± 0.086 (*n* = 41)	0.90 ± 0.21 (*n* = 41)	<0.001
Left anterior descending artery (*Z*-score)	0.379 ± 0.086 (*n* = 41)	0.91 ± 0.06 (*n* = 40)	<0.001
Right coronary artery (*Z*-score)	0.344 ± 0.076 (*n* = 41)	1.20 ± 0.18 (*n* = 41)	<0.001

^a^ From September 2021 to February 2022.

^b^ From March 2022 to September 2022.

## 4. Discussion

In this study we examined clinical outcomes including changes in coronary artery diameter and laboratory data in after COVID-19 infection in patients with a prior history of KD. We found that *Z*-score of the right coronary artery was slightly larger after COVID-19 infection, although the change was not large enough to be considered a coronary artery dilatation. We also found that increases in right coronary size was associated with a decrease in monocyte percentage after COVID infection, and was more likely to occur in patients who did not receive at least one dose of mRNA COVID-19 vaccination. It is also possible that these changes in RCA coronary artery diameter may also be the result of measurement imprecision or interobserver bias, which we have further discussed below.

In our study we found that right coronary artery *Z*-scores were statistically higher after COVID-19 infection in patients with a prior history of KD. Although symptoms of COVID-19 infection are primarily respiratory, cardiac complications during the acute phase include myocarditis, coagulation abnormalities, heart failure or arrhythmia (2). This may be in part due to the fact that angiotensin-converting enzyme 2 (ACE2), which is expressed the membrane of many cell types, including myocardiocytes, is a cellular receptor of SARS-CoV-2, and acts as an important cellular entry point for the virus (11). In adults, cardiovascular complications from COVID-19 infection are more prevalent among patients with a history of pre-existing cardiac disease (12).

In addition, we found that increases with RCA *Z*-scores were associated with a decrease in monocyte percentages 1–4 weeks after COVID-19 infection. In a study of 46 adults with COVID-19 infection, changes in monocyte populations were followed in the convalescent phase from 15 to 60 days after COVID-19 infection. The results showed that during this time period, there is a decrease in the absolute number inflammatory classical monocytes, and an increase in modulatory intermediate or non-classical monocytes. In addition, the researchers found that severe COVID-19 infection was associated with lower intermediate and non-classical monocyte numbers (13). It is possible that the KD patients in our study who had lower monocyte percentages 1–4 weeks after COVID-19 infection, may have had inadequate recruitment of immunomodulatory intermediate and non-classical monocytes, leading to increased cardiac inflammation and hence a greater increase in RCA *Z*-scores.

Vaccination against SARS-CoV-2 has been essential in curtailing the continued spread of the COVID-19 pandemic, and there is ample evidence that full immunization if effective in preventing severe disease (14). Less is known if vaccination confers protection against cardiovascular sequalae of COVID-19. In a Korean nationwide survey of COVID-19 vaccination outcomes found that patients who were fully vaccinated had a lower risk of acute myocardial infarction after COVID-19 infection (15). Similarly, in this study we found that in children with a prior history of KD, those who received at least one dose of mRNA COVID-19 vaccination were less likely to have increased RCA *Z*-scores after COVID-19.

There are several limitations to our study. For one, our subject number is quite low, with only 41 patients who fulfilled our entry criteria and were recruited in our study. In addition, only 9 patients included in our study had data regarding biochemistry values such as CRP, AST and ALT. Because of this, it is difficult for us to draw conclusions as to whether COVID-19 infection has an effect on these values in children with a history of KD. In addition, we found a few outliers within our data set by calculating the mean and standard deviation for both changes in RCA diameter and monocytes. Two outlier points were found to have a *Z*-score of higher than 3 (i.e., their value was 3 standard deviations higher or lower than the mean) (16). However, the removal of outliers did not change our results. After removal of the two outlier data points, changes in RCA diameter were still negatively correlated with changes in monocyte percentage, and achieved statistical significance. Therefore, we felt it was better to leave in the outliers because our data set was small to begin with. Moreover, because the regression models made available by the Taiwan Society of Cardiology were modeled on children less than 6 years of age, it may limit the accuracy of *Z*-scores in patient older than 6 years of age. To address this we recalculated the *Z*-scores of the RCA, LCA and LAD in children older than 6 years of age using the equations provided by Olivieri, which was derived from a set of 432 normal echocardiograms from healthy subjects age 0–20 years old (17). We found that *Z*-score values for LCA, LAD and RCA derived from the Taiwan Society of Cardiology all highly correlated with the *Z*-score values calculated using the Olivieri equation (Pearson's correlation 0.999, 0.999 and 0.988 respectively). Therefore, we opted to keep our original measurements for calculation. Another limitation could be that we could only find healthy controls that had received one echocardiograph within a time period and could not confirm whether they had been exposed to COVID-19 within our chart records. However, we feel that it is likely that many healthy controls in the post omicron wave group were exposed to COVID for the first time. According to publicly available data, the number of cumulative confirmed COVID cases stood at less than 1% of the total population at the beginning of 2023, and had ballooned to approximately 40% of the population by the end of 2023, while the actual percentage (including undocumented cases) is likely to be much higher (18).

It is important to note that these changes in RCA diameter could also be the result of measurement imprecision or interobserver variability. Furthermore, changes in coronary artery diameter, may also be the result of natural changes over time. In an interesting study which included 562 KD patients with coronary artery lesions received coronary angiography at least two points in time. The authors found that 15 of the patients had either new or expanding coronary artery lesions (19). A more recent study of 484 of KD patients who were followed over a period of 3 years found that 44 of them had progressive coronary artery aneurysms, with 7 developing giant coronary artery lesions, 16 developing medium-sized lesions, and 21 developing small-sized lesions (20). Additional studies in larger populations are needed in the future, and whether our results may be reproducible remains to be seen.

There is reason to believe that persistence inflammation after SARS-CoV-2 infection may be responsible for MIS-C in children. Failure of NK cells to adequately downregulate effector CD8+ T cells (i.e., NK cell dependent effector CD8+ T cell exhaustion, resulting in prolonged inflammation after SARS-CoV-2 infection has been proposed as a possible mechanism. In a study which examined blood RNA transcriptomes in patients with MIS-C and controls found that patients with MIS-C had lower NK cells numbers, together with lack of CD8+ T cell exhaustion appears to support this hypothesis (21). In addition, growing attention has been paid to the existence of COVID-19 symptoms which persist past the acute stage, a phenomenon termed “long COVID,” which may also stem from abnormal immunological response to viral infection (22). Taken together, the evidence suggests that patients with COVID-19 should receive long term follow-up. The results of our study also show that adequate follow-up for COVID-19 patients with a prior history of KD may be especially important.

## 5. Conclusion

This is the first to examine outcomes echocardiographic and laboratory outcomes of COVID-19 in children with a prior history of KD. We found that COVID-19 resulted in slightly higher RCA *Z*-scores within the first month after infection, although the degree of dilatation found was not large enough to be classified as a coronary artery aneurysm. While these changes could be the result of measurement imprecision or interobserver variation, due to the small scale of our study, further large scale or multicenter trials which examine the cardiac outcomes of COVID-19 infection in children with a prior history of KD are needed in the future.

## Data Availability

The raw data supporting the conclusions of this article will be made available by the authors, without undue reservation upon reasonable request.
